# Common Bed Bugs: Non-Viable Hosts for *Trypanosoma rangeli* Parasites

**DOI:** 10.3390/cells13242042

**Published:** 2024-12-11

**Authors:** Sanam Meraj, Phillip Phung, Kelvin Lau, Carl Lowenberger, Gerhard Gries

**Affiliations:** Department of Biological Sciences, Simon Fraser University, Burnaby, BC V5A 1S6, Canada; phillip_phung@sfu.ca (P.P.); kelvin_lau_4@sfu.ca (K.L.); clowenbe@sfu.ca (C.L.); gries@sfu.ca (G.G.)

**Keywords:** *Cimex lectularius*, *Trypanosoma rangeli*, Chagas disease, *Trypanosoma cruzi*, vector competence, immune response, parasite–host interactions, defensins, prolixicins

## Abstract

The hemoflagellate parasite *Trypanosoma rangeli* is transmitted by triatomine kissing bugs and may co-infect humans together with its Chagas disease-causing congener *T. cruzi*. Using real-time quantitative polymerase chain reaction (RT-qPCR) and antimicrobial assays, we studied (*i*) the temporal and spatial distribution of *T. rangeli* in common bed bugs, *Cimex lectularius*, following oral ingestion and hemocoelic injection of *T. rangeli,* and (*ii*) the immune responses of bed bugs induced by *T. rangeli* infections. Irrespective of infection mode, no live *T. rangeli* were present in the bed bugs’ hemolymph, salivary glands, or feces. On day 1 following infection, the bed bugs strongly upregulated the antimicrobial peptide CL-defensin. Following hemocoelic injection of *T. rangeli*, live parasites were absent in any bed bug tissues examined throughout the 10-day study period. The ingestion of *T. rangeli*-infected blood had no significant effect on bed bug survival. Our findings indicate that bed bugs disable the development of *T. rangeli* within their body, in stark contrast to triatomine kissing bugs, which allow the development and transmission of *T. rangeli*. Our findings help unravel the intricate relationships between bed bugs and trypanosomes, and they contribute to our understanding of vector biology.

## 1. Introduction

The hemoflagellate parasite *Trypanosoma rangeli* exhibits pathogenicity towards its insect hosts, but it appears non-pathogenic to humans [1,2,3,4,5,6]. Nonetheless, *T. rangeli* garners significant interest because it co-occurs in both vertebrate and invertebrate (insect) hosts with *T. cruzi,* the causal agent of Chagas disease. This co-occurrence complicates the diagnosis of Chagas disease, and it affects our epidemiological understanding of these parasites across Latin America [4]. *Trypanosoma rangeli* is transmitted by triatomine kissing bugs in the genus *Rhodnius* [2,4,7]. Kissing bugs are infected with *T. rangeli* when they ingest blood from infected vertebrates and wild and domesticated animals [2,4,7,8,9,10,11]. Within the gastrointestinal tract of kissing bugs, *T. rangeli* undergoes development and then migrates to the hemolymph, where it encounters the insects’ immune defenses. Despite these immune defenses, certain parasite strains proceed to the host’s salivary glands, where they develop into infective metacyclic trypomastigotes [2,4,7,8,9,10,11]. This salivary gland invasion by parasites is an obligatory step for transmission [12]. Parasitic infections can profoundly impair the development, reproduction, and survival of the insect hosts. Both *T. cruzi* and *T. rangeli* infections lower the survival of *Rodnius prolixus* hosts [6,13,14]. Intriguingly, however, *T. cruzi* infections of common bed bugs, *Cimex lectularius,* do not alter the bed bugs’ survival [15].

Common bed bugs are global blood-feeding pests of humans [16,17,18] and close phylogenetic relatives of kissing bugs [16]. Consequently, the potential for bed bugs to ingest human pathogens continues to be of significant concern and interest [18,19,20,21]. Many pathogens have been isolated from bed bugs [18,19,21,22], and some of them have been transmitted by bed bugs in laboratory settings [15,19,23,24]. In laboratory settings, bed bugs also transmitted the Y strain of *T. cruzi* through their feces [15,19,25,26], but in field settings, bed bugs are not known to transmit any strain of *T. cruzi, T. rangeli*, or other human pathogens. 

Even in their insect hosts, there are distinct host–parasite specificities. In only 2–50% of kissing bugs can *T. rangeli* cross the intestinal epithelium, enter the hemolymph, penetrate the salivary glands, and complete its development [1,3,27]. Each *Rhodnius* species seems to selectively transmit only certain strains of *T. rangeli* [28,29,30]. 

Insect immune responses to parasitic infections include the production of antimicrobial peptides (AMPs) and other immune factors [31,32,33] that curtail the development of parasites and limit their vectorial capacity [34,35,36]. Bed bugs express several AMPs [16,37,38,39,40], one of which, CL-prolixicin2, significantly reduced the survival and motility of *T. cruzi* parasites *in vitro* [38]. However, AMP expression by bed bugs in response to trypansome infections, including *T. rangeli* infections, have not been studied to date *in vivo*.

Here, we infected bed bugs with *T. rangeli* through oral ingestion and hemocoelic injection. We (1) investigated the development and the temporal and spatial distribution of *T. rangeli* within bed bugs and their excreta, (2) studied the expression of the CL-defensin and CL-prolixicin AMPs as a proxy for overall immune responses in the midgut and the RoB (rest of body containing bodies *minus* heads and midgut tissues) in bed bugs, (3) explored correlations between AMP expression levels and changes in antimicrobial activity in bed bug protein extracts of these tissues, and (4) investigated the effect of *T. rangeli* on bed bug survival. 

## 2. Materials and Methods

### 2.1. Laboratory Rearing of Bed Bugs 

Colonies of bed bugs were maintained as described [41]. Briefly, colonies were kept in the insectary of Simon Fraser University (SFU) at a temperature of ∼24 °C, ambient relative humidity, and a photoperiod of 14 h light to 10 h dark. Groups of 150 bed bugs were maintained in 50 mL glass jars fitted with a square of cardboard (2 cm × 2 cm) at the bottom and a strip of cardboard (2 cm × 4 cm) diagonally across the jar as walk-on substrates. Bed bugs in separate jars were fed on the forearm of a volunteer (Regine Gries) once every month. For feeding, jars were covered with fine mesh, inverted, and pressed against the forearm so that the bed bugs could feed through the mesh.

### 2.2. Bacterial Culturing and Quantification

Bacterial cultures of laboratory-propagated *Escherichia coli* K12/D31 and *Bacillus subtilis* (ATCC 6633) were used in the bed bug survival test and in antimicrobial activity assays. All cultures were maintained on Mueller Hinton (MH) media following standard microbiological procedures. Stock cultures were kept on MH agar plates stored at 4 °C and were propagated to fresh plates every 2–3 weeks. Prior to use in experiments, isolated colonies were obtained from stock cultures by streak-plating onto fresh plates and by incubating them overnight at 37 °C. From each streak plate, a single colony was selected and inoculated into 5 mL of MH broth, which was then grown in a 37 °C shaking incubator (Thermo Scientific MaxQ 3000; Thermo Fisher Scientific, Burnaby, BC, CA) at ~250 rpm either overnight for zone of inhibition (ZOI) assays or for 4–6 h to the log phase of growth for the bed bug survival tests. Bacterial concentrations in the broth cultures were quantified using a spectrophotometer (Shimadzu UV-2550; Sognsveien 70 A, 0855 Oslo, Norway), and a standard optical density (OD) curve was measured at OD600 to determine the number of colony-forming units (CFUs) per mL.

### 2.3. Maintenance and Propagation of T. Rangeli Parasites

*Trypanosoma rangeli* parasites [Tejera strain E1 Tocuyo, sub-strain JJ8/8 (ATCC 30033)] were purchased from the Cedarlane supplier of the ATCC (American Type Culture Collection; Manassas, VA, USA) and maintained using the ATCC’s standard procedure. This strain is well-characterized and widely used in infectious disease research, is known to infect the salivary glands of *Rhodnius prolixus* kissing bugs (primary *Trypansoma* vectors), and has extensively been studied as a model organism for understanding vector competence. 

Parasites were maintained in a diphasic *Trypanosome* medium (ATCC medium 431) and incubated at 25 °C under axenic conditions. The diphasic medium consisted of (*i*) a solid-phase blood agar medium (3.0 g beef extract, 5.0 g peptone, 8.0 g NaCl, 15.0 g agar, and 1.0 L distilled water) supplemented with 30% (by volume) defibrinated rabbit blood (HemoStat Laboratories; Dixon, CA, USA) and (*ii*) a liquid-phase Locke’s solution (8.0 g NaCl, 0.2 g KCl, 0.2 g CaCl_2_, 0.3 g KH_2_PO_4_, 2.5 g glucose, and 1.0 L distilled water) over the top of each blood agar slant. New cultures of *T. rangeli* trypomastigotes were established by inoculating 50 µL of the liquid supernatant from a previously cultured trypomastigote suspension into a fresh Trypanosome-specific medium. Propagations were performed every 3–4 days. This procedure kept the parasites in the log phase of growth. Parasites were quantified in a Neubauer chamber.

### 2.4. Infection of Bed Bugs with T. rangeli through Ingestion or Hemocoelic Injection

Adult male bed bugs, blood-starved for > 20 days, were subjected to two modes of infection with *T. rangeli*: (1) ingestion of *T. rangeli*-infected blood and (2) intrathoracic (hemocoelic) injection. The parasite concentrations were adjusted using a hemocytometer.

For the oral ingestion of *T. rangeli*, 100–200 starved male bed bugs in the treatment group (1 bed bug per replicate) fed on defibrinated rabbit blood containing approximately 1 × 10^7^ parasites/mL in their log growth phase using a water-jacketed membrane feeder (Thermo Fisher Scientific Isotemp 2150 B14, USA) set to 37 °C, with stretched-out parafilm as the membrane. The 100–200 bed bugs in the control group (1 bed bug per replicate) fed on sterile blood. All bed bugs replete with blood were isolated and kept in glass jars for subsequent analyses.

Similarly, for hemocoelic injection, 100–200 starved male bed bugs in the treatment group (1 bed bug per replicate) were injected with a 0.5 μL PBS (phosphate-buffered saline) solution containing approximately 1 × 10^5^ parasites/mL, whereas the bed bugs in the control group (1 bed bug per replicate) were injected with 0.5 μL PBS. Injections were performed using a precision Hamilton syringe (Sigma-Aldrich, Saint Louis, MO, USA) directly injected into the bed bugs’ hemocoel. To capture the acute phase of infections and to track the bed bugs’ innate immune responses, bed bug hemolymph was collected 1 h after the injection and on days 1, 4, and 7 thereafter. For each time point, the hemolymph was withdrawn from five separate bed bugs, each representing a replicate.

### 2.5. Confirmation of Infection and Quantification of Parasites

The presence and abundance of parasites in bed bugs were tracked immediately after their ingestion by bed bugs and on subsequent days 1, 4, 7, 10, 20, and 30. For each time point, 10 bed bugs were individually analyzed for the presence of *T. rangeli* in the hemolymph, the anterior midgut, the combined posterior midgut and hindgut, salivary glands, and feces. 

For the analyses, bed bugs were surface-sterilized in 70% ethanol, followed by PBS washes to eliminate any ethanol traces. Subsequently, each bed bug was dissected under a stereoscopic microscope (Carl Zeiss Stemi 2000-C, Jena, Germany) to isolate the midgut, salivary glands, hemolymph, and feces. To avoid any potential cross-contamination of samples (particularly the midgut samples), they were triple-washed with PBS. The midgut was divided into two segments: the anterior midgut and the posterior midgut/rectum. These midgut segments were then placed in tubes containing 50 μL of sterile water for homogenization. The presence or absence of live parasites in all samples at each time point was recorded. 

Salivary glands of bed bugs were placed on a glass slide and ruptured using dissection scissors. The presence of live parasites within these salivary glands was observed under a phase-contrast oil immersion microscope at magnifications of 400× and 1000×. To extract the hemolymph, each bed bug’s extremity was carefully removed, and the abdomen was gently compressed 10 s to induce hemolymph flow, 0.5 μL of which was collected in an Eppendorf microtube, pre-lined with PBS, to achieve a 50-fold dilution. Both diluted and undiluted samples were examined under a light microscope using phase-contrast settings in separate experiments. Furthermore, a section of the bed bug’s body was pierced with a needle for further analysis of the hemolymph under a phase-contrast microscope. 

Fecal matter was obtained by exerting pressure on the bed bug’s abdomen. Excreted fecal matter (0.5–1.0 μL per bed bug) was diluted in PBS (50 μL) and observed under a hemocytometer with a light microscope equipped for phase-contrast viewing.

To determine how *T. rangeli*-injected bed bugs (see above) cope with these parasites, 0.5 μL of hemolymph was withdrawn (see above), diluted in PBS (20 μL), and then observed under a microscope (see above) for the presence of live or dead *T. rangeli*. Moreover, the midgut and salivary glands were also examined (see above).

### 2.6. Testing for the Presence of Parasites Using Polymerase Chain Reaction (PCR) Analysis 

Immediately after bed bugs had ingested *T. rangeli*-infected blood, and 1, 4, 7, 10, and 17 days later, they were analyzed for the presence of *T. rangeli* DNA. Each bed bug’s body surface was sterilized with 70% ethanol and washed twice with PBS. For each time point, three bed bugs in each of the three replicates were analyzed by PCR. DNA of whole bed bugs was extracted using the DNeasy blood and tissue extraction kit (QIAGEN, Valencia, CA, USA). DNA of *T. rangeli* was detected by PCR. The following pair of forward (F) and reverse (R) primers—designed to amplify a 105 bp fragment of the *T*. *rangeli* annotated *KMP-11* gene—was used: KMP84_F: GAAGTTCTTTGCGGACAAGC and KMP188_R: TTGAACTTGTCGGTGTGCTC [42]. PCRs were run in 25 μL reaction mixtures containing 12.5 μL of Kodaq mastermix, 0.5 μL of forward and reverse primers, and 100–200 ng of extracted bed bug DNA from bed bugs fed either sterile blood or *T. rangeli*-infected blood. For positive controls, DNA was extracted from the *T. rangeli* culture, whereas for negative controls, UltraPure Distilled Water or uninfected bed bug DNA replaced the infected bed bug DNA. A PTC-200 Peltier Thermal Cycler (MJ Research, Saint-Bruno-de-Montarville, QC, Canada) was used for all PCR amplifications. The program was set to 94 °C for 2 min, followed by 40 cycles each at 94 °C, 52 °C, and 72 °C and a final extension step at 72 °C for 5 min. The presence of the expected band size at approximately 100 base pairs (bps) and the success of the PCR amplification were confirmed on a 0.7% agarose gel with a 1 kb Plus Opti DNA Marker (ladder) (Applied Biological Materials, Richmond, BC, Canada). PCR amplicons were purified using the QIA PCR & Gel Cleanup Kit (Qiagen, Venlo, The Netherlands). Amplicons were sequenced (Genewiz, South Plainfield, NJ, USA), and the Basic Local Alignment Search Tool (BLAST) [43] was used to compare the sequenced region of individual isolates with known sequences.

### 2.7. Bed Bug Survival After Ingestion of T. rangeli

As many as 174 adult male bed bugs (starved 28–30 days) were used to assay the effect of *T. rangeli* infection on bed bug survival after ingestion of sterile or *T. rangeli*-infected blood. Bed bugs were randomly assigned to one treatment group and two control groups. Each group was kept in a separate jar and fed using the water-jacketed membrane feeder as described. As a negative control group, 24 bed bugs were each fed sterile defibrinated rabbit blood. As a positive control group, 50 bed bugs each were fed defibrinated blood inoculated with *E. coli* at 10^7^ CFU/mL. As the treatment group, 50 bed bugs were each fed defibrinated rabbit blood inoculated with *T. rangeli* at a concentration of 10^7^ parasites/mL. Immediately after feeding, any bed bugs not engorged with blood were excluded from subsequent analyses. Every day for 22 days, dead bugs in each group were removed and recorded.

### 2.8. Zone of Inhibition (ZOI) and Minimum Inhibitory Concentration (MIC) Assays with Midgut and RoB Tissues of T. rangeli-Fed Bed Bugs

The antimicrobial activity of the midgut and RoB samples, obtained from bed bugs fed *T. rangeli*-infected blood, was tested in zone of inhibition (ZOI) assays. Samples were taken from bed bugs immediately after feeding from the water-jacketed membrane feeder and 1, 4, 7, and 10 days later. Each biological replicate consisted of 10 bed bugs, with three replicates run for each time point.

For protein extractions, bed bugs were washed in sterile PBS solutions and were dissected and homogenized in a mixture of protein extraction buffer (90 µL; 0.1 % triton x-100, 100 mM NaCl, and 50 mM Tris, pH 7.0) and proteinase inhibitor (10 µL). Homogenates were then centrifuged (Hermle Labnet Z 252 MK D-7209 Gosheim, Labnet International, Inc., Wehingen, Germany) to remove bulk tissue, and the remaining protein extracts were kept at −20 °C for <2 weeks prior to assays. Protein concentrations of samples were measured using a Bradford Assay kit (Bio-Rad Laboratories, 500-0001; Mississauga, ON, Canada) following recommended procedures. All protein extraction samples were diluted to the same 1.0 µg/µL concentration for use in ZOI assays.

Zone of inhibition (ZOI) assays were run according to the Kirby–Bauer disk diffusion method. Briefly, *E. coli* and *B*. *subtilis* cultures were grown separately in Mueller–Hinton (MH) broth and diluted in sterile water to final concentrations of 10⁶ and 10⁷ CFU/mL, respectively. These bacterial suspensions were then evenly aliquoted onto 4 mm deep MH agar plates. Sterile 6 mm filter paper disks were placed in a ring formation on each agar plate, and 10 µL of protein extract, containing 10 µg of protein, was applied to each disk. Every agar plate also received a filter paper disk treated with sterile water as a negative control, and a disk treated with lysozyme (Novagen^®^ 71412) or Ampicillin (Product No. A9393-5G) served as a positive control. All agar plates were incubated for 12–24 h at 37 °C before measuring the ZOIs around the filter paper disks. The plates were run in duplicate to ensure the reproducibility of results. ZOI measurements were taken under a dissecting microscope using a clear plastic ruler. A ZOI was defined as a zone without any growth of a bacterial lawn. Any zone with even a single bacterial colony present was considered a negative result in the context of this experiment. Positive ZOIs were measured twice, perpendicularly, with the mean of both measurements taken as the result.

The minimum inhibitory concentration (MIC) is defined as the minimum protein concentration at which no bacterial growth is observable [39,44]. MICs were determined using the broth microdilution method in 96-well microtiter plates with an assay volume of 100 μL in each well. Briefly, 2-fold serial dilutions of bed bug protein extracts (concentration range: 0.0008 to 1.0 μg/μL) were prepared [39,44], and a 50 μL aliquot of each concentration was transferred to a plate well. Overnight bacterial cultures, regrown to the log phase of growth, were diluted to a final concentration of ~5 × 10^5^ CFU/mL in a non-cation-adjusted MH broth (Difco; BD, Franklin Lakes, NJ, USA), and a 50 μL aliquot was mixed with the content of each well. After overnight incubation at 37 °C, bacterial growth was assessed by measuring light absorbance at 600 nm (Shimadzu UV-2550 spectrophotometer). All dilutions of extracted proteins were tested in two technical replicates and three biological replicates. Mueller–Hinton broth and Ampicillin (Product No. A9393-5G) served as negative and positive controls, respectively. 

### 2.9. Real-time Quantitative Polymerase Chain Reaction (RT-qPCR) Experiment for CL-Prolixicin and CL-Defensin Expression

Time-specific expression levels of CL-prolixicin and CL-defensin in the midgut and RoB tissues of bed bugs in response to *T. rangeli* infection were measured using RT-qPCR immediately after ingestion of *T. rangeli*-infected blood and 1, 4, 7, 10, 13, and 17 days post-ingestion. Total RNA was extracted using the TRizol reagent (Invitrogen, Carlsbad, CA, USA) as per the manufacturer’s protocol. Sample concentrations were quantified on a Nanodrop 1000 spectrophotometer v. 3.7 (Thermo Fisher Scientific, Altham, MA, USA). First-strand cDNA synthesis was performed in 20 µL reaction mixtures containing 2.0 µg total RNA using a modified oligo dT primer (MgdT) with a OneScript cDNA Synthesis Kit (Applied Biological Materials). Template cDNA samples were amplified using CL-prolixicin1 (LOC106664366) primers (F-5-ACG GAC CTA ACC CTT CCA GAT-3; R-5-GAT CCC GTA CAT TCT GTG CC-3) and CL-defensin3 (LOC106661793) primers [37,38], and were compared against both the background housekeeping gene RPL18 primers (F-5-AAA GGC ACG GTT ACA TCA AAG GTG-3; R-5-TAG TCT TGA ACC TAT AGG GGT CCC-3) and the sample prepared immediately after feeding for the time-dependent analysis. Each PCR well contained 5 µL of PerfeCTa SYBR Green Super Mix (Quanta Biosciences, Beverly, MA, USA), 2 µL of sterile H_2_O, 0.5 µL of each required primer (0.1 µM), and 2 µL of the template cDNA diluted 1:10 times with sterile RNAse-free water. RT-qPCR was performed using a LightCycler96 thermal cycler (Roche Diagnostics, Penzberg, Germany). For each run, samples were first pre-denatured for 3 min at 98 °C. Then, samples were cycled 30 times through 10 s of denaturation at 98 °C, 30 s of annealing at 60 °C, and 153 s of extension at 72 °C. A final extension was run at the end of the cycle for 120 s at 72 °C. The resulting PCR data were quantified and analyzed using the 2^ΔΔC(T)^ method [45,46].

### 2.10. Statistical Analysis

Based on data distributions and the number of treatments, data were analyzed with one-way ANOVA, Student’s *t*-test, the Kruskal–Wallis test, or the Mann–Whitney test, using GraphPad Prism 8 software. Statistical significance was set to *p* < 0.05. Bed bug survival analyses used the Kaplan–Meier method (GraphPad Prism (version 8.0.2) software, manufactured by GraphPad Software Inc., San Diego, CA, USA).

## 3. Results 

### 3.1. Detection of T. rangeli in Bed Bug Tissues Following Ingestion or Hemocoelic Injection of T. rangeli

Irrespective of the infection mode (oral ingestion or hemocoelic injection), no live *T. rangeli* were observed in the salivary glands, hemolymph, or feces on any day during the 17-day post-infection period (Table 1) or the 45-day post-infection period. As *T. rangeli* was absent in the salivary glands, its transmission through feeding bites was not possible.

**Figure 1:**
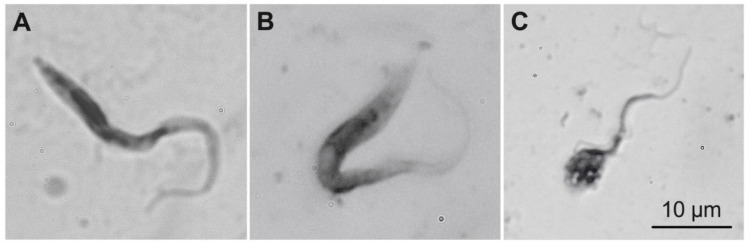
Life stages of *Trypanosoma rangeli* found in the anterior midgut of the common bed bug, *Cimex lectularius*: Epimastigotes (**A**); intermediate forms (**B**,**C**). Cells were stained with Giemsa and visualized using oil emersion on a compound light microscope (×1000 magnification; scale: 10 μm).

On days 1, 4, and 7 following ingestion of *T. rangeli*-infected blood, live parasites were present in the anterior midgut of bed bugs but were absent in the posterior midgut. Similarly, *T. rangeli* DNA was found in all samples tested on days 1 and 4 but not on day 7. On days 7 and 10, non-viable parasites were present in the posterior midgut of bed bugs, and different forms of *T. rangeli* were present in the anterior midgut (Figure 1).

When the bed bugs were subjected to intrathoracic injection of *T. rangeli*, live parasites were absent in any bed bug tissues examined throughout the duration of the study. However, non-viable parasites were detected in the anterior midgut of bed bugs on day 1 post-injection, implying that the parasites could enter the anterior midgut but did not survive there. 

### 3.2. Antimicrobial Activity of T. rangeli-Fed Bed Bugs Against B. subtilis

**Figure 2:**
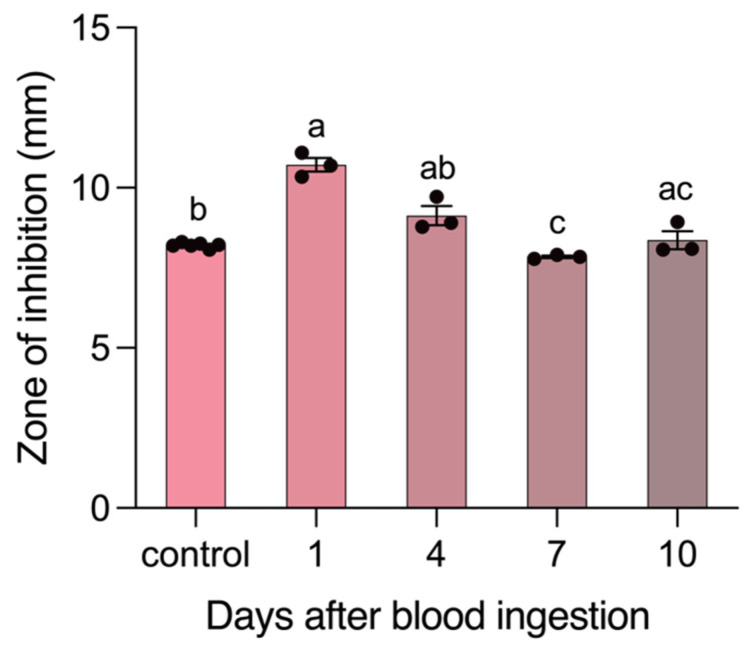
Results of the zone of inhibition (ZOI) test (three to five replicates) run to measure antibacterial (AB) activity expressed in midgut extracts of adult male bed bugs 0–10 days after an immune challenge. AB activity was tested against the bacterium *Bacillus subtilis* (10^7^ cells/mL) and quantified as the zone of inhibited bacterial growth around a piece of filter paper treated with midgut protein extracts from bed bugs that had ingested sterile blood (control) or blood containing the parasite *Trypanosoma rangeli.* Bars represent the mean zone of inhibition ± 95% confidence intervals. Means were compared using the unpaired Student’s *t*-test. Different letters on bars indicate significant differences.

No RoB protein extracts, from control or *T. rangeli*-exposed bed bugs, inhibited the growth of *B. subtilis* and *E. coli*. However, the midgut extracts of bed bugs that had ingested *T. rangeli*-infected blood inhibited *B. subtilis* growth (Figure 2). When midguts were extracted 1 day after bed bugs had ingested *T. rangeli*-infected blood, these extracts were significantly more inhibitory to *B. subtilis* than the midgut extracts of control bed bugs that had ingested sterile blood. However, this inhibitory effect subsided between days 4 and 7 (*p* < 0.0001; Figure 2). Similarly, in MIC assays with bed bug RoB and midgut extracts, the strongest inhibition of *B. subtilis* was observed when extracts were prepared 1 day after bed bugs had ingested *T. rangeli*-infected blood (Table 2). 

### 3.3. RT-qPCR Test for CL-Prolixicin and CL-Defensin Expression

**Figure 3:**
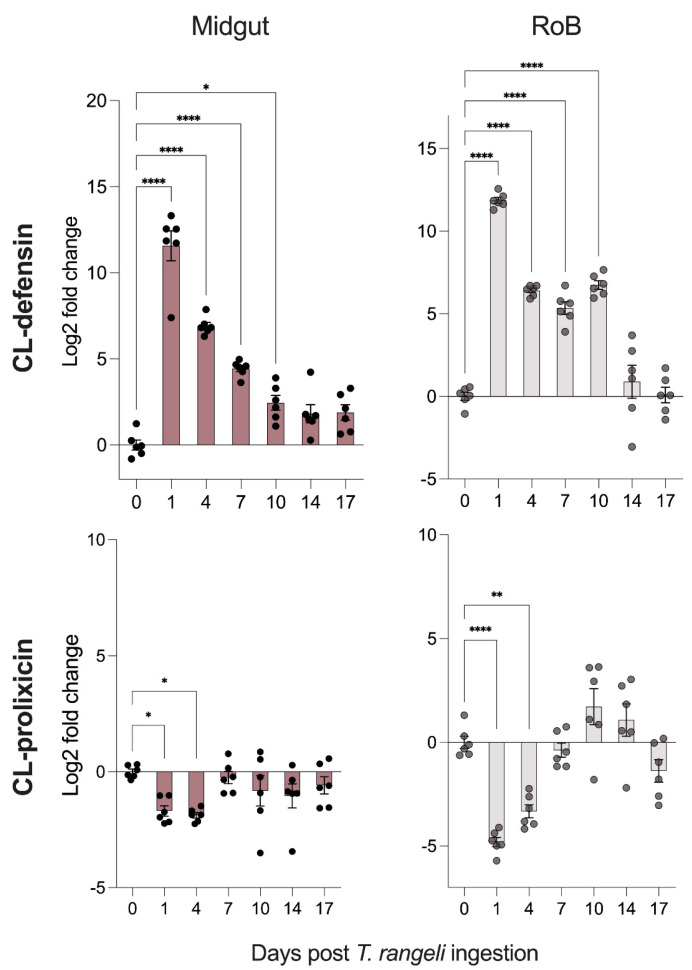
Time-dependent expression of CL-defensin mRNA (LOC106661793) and CL-prolixicin mRNA (LOC106664366) in bed bugs after *Trypanosoma rangeli* ingestion. Expression levels of both antimicrobial peptide genes were quantified using the 2^−ΔΔC(T)^ method [45,46]. Bars represent the mean transcript levels ± 95% confidence intervals. Means were compared using the unpaired Student’s *t*-test (* *p* < 0.05, ** *p* < 0.01, **** *p* < 0.0001).

The ingestion of *T. rangeli* affected the expression of CL-defensin and CL-prolixicin. On day 1 following ingestion, CL-defensin was upregulated 300- to 5000-fold in both the midgut and RoB samples (*p* < 0.0001 each; Figure 3), but CL-defensin expression strongly subsided during days 1–17 after the infection. In contrast, CL-prolixicin in the midgut samples was downregulated on all days following infection and in the RoB samples on most days except days 10 and 14 (Figure 3). In the RoB samples, CL-prolixicin was downregulated 30-fold (*p* < 0.0001) and 10-fold (*p* < 0.001) at days 1 and 4, respectively (Figure 3). 

### 3.4. Bed Bug Survival After Ingestion of T. rangeli

**Figure 4:**
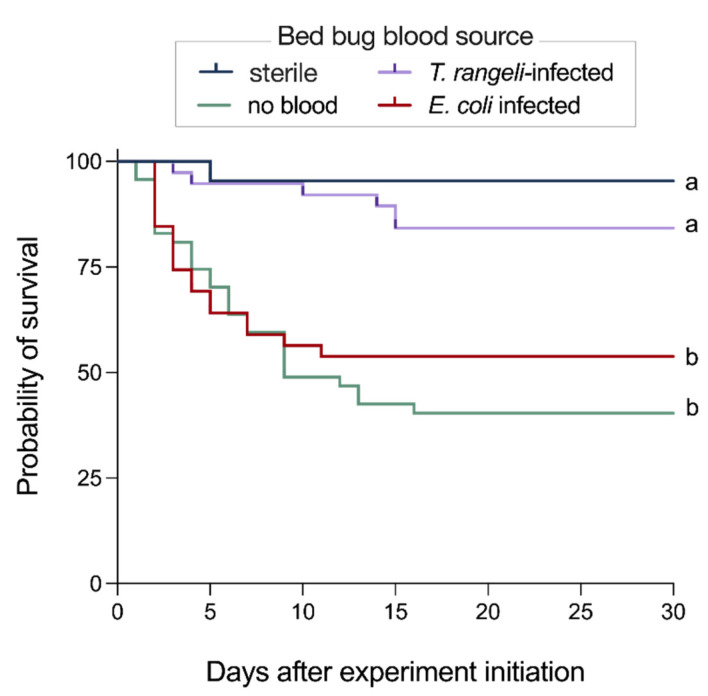
Kaplan–Meier plots depicting the survival of bed bugs that were starving (green line), had ingested sterile blood (black line), or had ingested blood infected with either the parasite *Trypanosoma rangeli* (purple line) or the bacterium *Escherichia coli* (red line). Lines marked with different letters are statistically different (*p* < 0.05). Note: Bed bugs that ingested sterile blood or blood infected with *T. rangeli* had the same probability of survival.

The ingestion of either sterile blood or *T. rangeli*-infected blood had no significant effect on bed bug survival (Figure 4). However, bed bugs that ingested *E. coli*-infected blood had significantly lower survival than bed bugs that ingested either *T. rangeli*-infected blood (χ^2^ = 10.67, df = 1, *p* = 0.0011) or sterile blood (χ^2^ = 10.79, df = 1, *p* = 0.0010). Furthermore, bed bugs that had ingested *E. coli*-infected blood, or that were starving, had equally low survival, lower than that of bed bugs that had ingested *T. rangeli*-infected blood (χ^2^ = 17.96, df = 1, *p* < 0.0001) or sterile blood (χ^2^ = 16.38, df = 1, *p* < 0.0001; Figure 4). 

## 4. Discussion

We studied the effects of *T. rangeli* infection mode—oral ingestion and hemcoelic injection into bed bugs—on both the parasites and their bed bug hosts. Specifically, we investigated the (*i*) temporal dynamics and spatial distribution of *T. rangeli* within bed bug tissues, (*ii*) the expression of two AMPs in response to *T. rangeli* infection, (*iii*) the antibacterial activity of extracts from tissues of *T. rangeli*-infected insects, and (*iv*) the pathogenicity of *T. rangeli* on its bed bug host.

Following the ingestion of *T. rangeli*-infected blood by bed bugs, *T. rangeli* first colonized the anterior midgut but was not detectable in any tissue, the hemolymph, or feces of bed bugs at, or after, day 10 post-ingestion. That *T. rangeli* was absent in these tissues is likely due to the bed bugs’ immune responses or to an inadequate adaptation of *T. rangeli* to bed bugs as host insects. The presence of *T. rangeli* only in the midgut of bed bugs contrasts with the distribution of *T. rangeli* in various tissues of the related kissing bugs, indicating disparate physiological environments in bed bugs and kissing bugs [2,7,11,47]. The success of *T. rangeli* and *T. cruzi* varies significantly with both the parasite strain and the parasite host, revealing differential interactions between the insect vector and the parasite strain it transmits [28,30,48]. Not all strains are able to complete their life cycle in certain hosts or to achieve transmission by their hosts [28,30,48].

Insect vectors ingest *T. rangeli* during blood meals from infected vertebrates. Unlike *T. cruzi*, which remains exclusively within the insect gut, *T. rangeli* penetrates the gut epithelium of its insect host and migrates through the hemocoel to the salivary glands [2,7,11,47]. In the anterior midgut, *T. rangeli* transforms into epimastigotes, then enters the hemocoel and finally transforms into infective trypomastigotes within the salivary glands [2,7,11,47]. That salivary glands of *Rhodnius* kissing bugs become infected with *T. rangeli* reveals the parasites’ ability to survive the hosts’ immune responses in the hemocoel—an ability not shared with *T. cruzi* [2,7,11,47].

In contrast, *T. cruzi* ingested by blood-feeding kissing bugs transforms into epimastigotes in the posterior midgut, proliferates and transforms into metacyclic trypomastigotes in the rectum, and is excreted together with feces that may contaminate insect bite wounds inflicted on humans [11,30,49,50,51,52,53]. When *T. cruzi* is injected directly into the kissing bugs’ hemocoel, the parasite is eliminated by the insects’ immune system [11,30,54,55], revealing different survival strategies of *T. rangeli* and *T. cruzi* and different host–parasite interactions.

That *T. rangeli* was absent in bed bugs shortly after oral ingestion or hemocoelic injection demonstrates that the immune system of bed bugs effectively eliminated these parasites or that no triggers to establish and develop in bed bugs were found. As no parasites were present in the bed bug hemolymph after the ingestion of *T. rangeli*, it follows that the bed bug gut serves as an immune barrier that prevents *T. rangeli* from entering the bed bug hemocoel. The fact that *T. rangeli* did not survive when injected into the bed bug hemolymph is further evidence for the bed bugs’ adept immune system that is capable of efficiently eliminating parasites. The bed bug immune responses following parasite infections include the upregulation of AMPs that play instrumental roles in suppressing parasite infections [5,8,34,56]. 

In response to *T. rangeli* ingestion, the bed bugs expressed CL-defensins and CL-prolixicins. Specifically, CL-defensins were upregulated in both the midgut and the RoB, with peak expression levels occurring 1 day after infection and elevated expression sustaining for up to 10 days. Conversely, the expression of CL-prolixicins was downregulated at first and then returned to baseline levels. In triatomines, immune defenses against *T. rangeli* include (*i*) activation of the Toll and IMD pathways that regulate AMP expression, (*ii*) modulation of gut microbiota, (*iii*) enzymatic activity of lysozymes and the prophenoloxidase (PPO), and (*iv*) other processes involving galactose-binding lectins and eicosanoid signaling pathways [2,3,5,47,49,56,57]. In bed bugs, we laid the foundation for understanding the tissue-specific elimination of *T. rangeli* parasites as well as the tissue-specific activation of the Toll and IMD pathways. Pathway activation was evidenced by downstream expression of the pathway effector AMPs defensins and prolixicins. As previously shown, CL-defensins and CL-prolixicins are upregulated in both the midgut and RoB of bed bugs in response to the ingestion and injection of *B. subtilis* and *E. coli* [37,38]. These changes in bed bug AMP expression mirror those observed in *R. prolixus* after *T. rangeli* infection. Much like the expression pattern of CL-defensin in bed bugs, Defensin C in kissing bugs was significantly upregulated in both the anterior and posterior midgut 1 day post-infection with *T. rangeli* [5], revealing an immediate immune response to the infection, which returned to baseline levels at day 7. In contrast, expressions of lysozyme B and prolixicin in kissing bugs were significantly reduced in the anterior and posterior midgut at day 1 and 7 post-infection [5]. It is noteworthy that the various strains of *T. rangeli* have different (mainly downregulating) effects on the immune system of triatomines, and that equivalent effects of these *T. rangeli* strains on the immune responses of bed bugs have yet to be investigated.

Future research on bed bugs should build upon the findings of this study, exploring immune responses observed in *T. rangeli*-infected triatomines, such as galactose-binding lectins, eicosanoid signaling, and microbiota modulation, in the context of bed bugs. Such studies would help determine comparable and unique immune responses by bed bugs infected with *T. rangeli*. Moreover, homologues of PPO (PPO6: LOC106662258, LOC106673360, LOC106673564, and LOC106673939) and of lysozymes (LYSC4A: LOC106663584, LOC106663588, LOC106669094, and LOC106673043; lysozyme-like: LOC106663700, LOC16666694, and LOC106667626)—which we have previously shown to be involved in the immune responses of bed bugs to bacteria [40]—should be fully characterized in the context of *T. rangeli* infections. Potential findings would facilitate further exploration (e.g., transcriptomic analyses) of immune pathways in bed bugs and would help unravel the complexity of host–parasite interactions in non-traditional vectors.

Trypanosome infections challenge the immune system of insect hosts and modulate the hosts’ survival and reproduction [15,53,58]. The effects of *T. rangeli* and *T. cruzi* infections vary both between and within host species [15,53,58]. When bed bugs ingested *T. rangeli* (this study) and *T. cruzi* (Arequipa TC-35 strain) [15], their survival was not affected. The bed bugs’ resilience might be attributed to their immune responses—the transient upregulation of AMP genes and the elimination of *T. rangeli* parasites soon after their ingestion. Conversely, studies on triatomine bugs have shown that infection with the *T. rangeli* CHOACHI strain and various *T. cruzi* strains can delay development, reduce survival and reproductive rates, and alter other phenotypic traits [6,13,14]. However, some studies report no significant effects [59,60]. Factors such as insect age, sex, specific *T. cruzi* strains, and environmental conditions, including temperature, have also been shown to influence life history traits in *T. cruzi*-infected triatomines [48,61,62].

## 5. Conclusions

Our findings contribute to a deeper understanding of vector–parasite interactions, emphasizing the complexity of these relationships and the importance of considering both physiological factors and immune responses of host insects. Our findings also highlight the need for continued studies that investigate the genetic and molecular bases of vector competence and the potential of insects that are deemed incompetent vectors—such as bed bugs—to contribute to the transmission of pathogens. In this study, *T. rangeli* seemed unable to complete its life cycle within bed bugs. These data align with previous conclusions that bed bugs are incompetent vectors of *T. rangeli* in field settings. Further research is needed to explore the mechanisms through which bed bugs eliminate *T. rangeli,* thereby affecting its transmission dynamics. The apparent inability of bed bugs to transmit parasites to vertebrate hosts remains an area of active research, requiring an interdisciplinary approach that combines molecular biology, immunology, and behavioral and community ecology.

## Figures and Tables

**Table 1:** Presence/absence of *Trypanosoma rangeli* parasites in various tissues of the common bed bug, *Cimex lectularius,* following oral ingestion of *T. rangeli*-infected blood or hemocoelic injection of *T. rangeli.* Data represent the number of bed bugs (out of 10) that contained live parasites. An asterisk (*) indicates the presence of non-viable (dead) parasites.

Oral Ingestion Experiment						
Days Post-Infection	Salivary Gland	Anterior Midgut	Posterior Midgut	Hemolymph	Feces	PCR of Whole Body
1	0/10	(10/10)	(0/10)	(0/10)	(0/10)	(3/3)
4	0/10	(10/10)	(0/10)	(0/10)	(0/10)	(3/3)
7	0/10	(4/10)	(0/10)	(0/10)	(0/10)	-
10	0/10	(0/10)	(0/10)	(0/10)	(0/10)	-
17	0/10	(0/10)	(0/10)	(0/10)	(0/10)	-
**Hemocoelic injection experiment**						
**Days post-infection**	**Salivary gland**	**Anterior midgut**	**Posterior midgut**	**Hemolymph**	**Feces**	
1	(0/10)	(0/10)	(0/10)	(0/10) *	(0/10)	
4	(0/10)	(0/10)	(0/10)	(0/10) *	(0/10)	
7	(0/10)	(0/10)	(0/10)	(0/10)	(0/10)	

**Table 2:** Minimum inhibitory concentration (MIC; lowest concentration at which no visible growth of the bacterium *Bacillus subtilis* occurred) from protein extracts obtained from the midguts and the rest of body tissues (RoB; bed bug bodies *minus* the heads and midgut tissues) of male bed bugs on days 1, 4, 7, and 10 after they had ingested blood containing the parasite *Trypanosoma rangeli.* Ingestion of sterile blood served as the control; DAI = day after ingestion.

	MIC (µg/µL) of Antibacterial Agents ^1^				
	Control ^2^	Parasite Ingestion			
	DAI = 1	DAI = 1	DAI = 4	DAI = 7	DAI = 10
Midgut	1	0.02	1	1	1
RoB	0.02	0.004	0.5	0.5	0.5

^1^ Data are based on 3 biological replicates and 2 technical replicates. ^2^ Bed bugs 1 day after ingesting sterile blood; note: data for control bed bugs on days 1, 4, 7, and 10 after ingesting sterile blood are not shown.

## Data Availability

All data generated or analyzed during this study are included in this article and Supplementary Materials.

## Supplementary Materials

The following supporting information can be downloaded at: https://www.mdpi.com/article/10.3390/cells13242042/s1.
